# Deep Brain Stimulation for Chronic Pain: Time to Reconsider the Skeptical Attitude?

**DOI:** 10.3390/brainsci10110772

**Published:** 2020-10-23

**Authors:** Konstantin V. Slavin, Emil D. Isagulayn, Dzhamil A. Rzaev

**Affiliations:** 1 Department of Neurosurgery, University of Illinois at Chicago, Chicago, IL 60612, USA; 2 Department of Functional Neurosurgery, Federal State Autonomous Institution, N.N. Burdenko National Scientific and Practical Center for Neurosurgery of the Ministry of Healthcare of the Russian Federation, 125047 Moscow, Russia; emisagulyan@gmail.com; 3 Federal Center of Neurosurgery, 630087 Novosibirsk, Russia; jamilrzaev@gmail.com; 4 Institute of Medicine and Psychology, Novosibirsk State University, 630090 Novosibirsk, Russia

Despite continuous advancements in systematic treatment of chronic pain there is still a subset of clinical conditions where the standard medical and surgical approaches are not uniformly effective. Moreover, in addition to these relatively rare but remarkably treatment-refractory diagnoses (post-stroke pain, brachial plexus avulsion pain, spinal cord injury pain, anesthesia dolorosa, etc.), there are many other, much more prevalent, conditions (persistent spinal pain syndrome (previously referred to as failed back surgery syndrome), painful peripheral neuropathy, complex regional pain syndromes, etc.) where even a small percentage of treatment-refractory patients constitutes a large group of chronic pain sufferers.

The treatment algorithms for these patients include gradual escalation of various modalities, usually chosen based on efficacy and safety, with treatment invasiveness being interpreted as direct measure of surgical risks and complications. The latter argument (on significant treatment-related risks) has been a deterrent for widespread acceptance of certain surgical interventions and a major source of reluctance for many pain-treating specialists and patients alike. This is mainly concerned the surgical neuromodulation of cerebral targets—the deep brain stimulation (DBS) and motor cortex stimulation (MCS). However, despite all obstacles (off-label status, significant device-related costs, skepticism among implanters), the worldwide experience with cerebral continues to grow.

The article by the neurosurgical team from Oxford [1] provides an excellent overview of the topic—going over pros and cons of each modality, their procedural details, target-specific limitations and comparative efficacy. Most importantly, it offers a rationale for future studies and presents a background for determination of optimal indications, individualized targets and criteria for success.

Much has been written on DBS for pain; the fascinating history behind this remarkable procedure is, in essence, a reflection of the entire modern functional neurosurgery: the innovation driven by need and purpose, careful observation and continuous advancement based on countless trials and errors, and gradual expansion of both targets and indications based on multiple enthusiastic centers of excellence.

DBS for pain remains an underused modality. While widely used for movement disorders [2,3] and under scrutiny for psychiatric indications [4,5,6], DBS has been overshadowed in the pain arena by spinal cord stimulation [7,8,9], peripheral nerve stimulation [10,11] and, to lesser extent, motor cortex stimulation [12,13]. However, the worldwide experience indicates that none of these procedures work every time and, therefore, there is a great need in maintaining DBS as an option for both common and uncommon indications. 

In times of serious societal concern about opioid epidemics, there is now more than ever a need in critical re-evaluation of surgery for pain, particularly when it comes to non-destructive interventions with decades of clinical experience. As the matter of fact, we have recently suggested a revision of the World Health Organization ladder approach to the pain management in favor of considering surgery earlier in the treatment algorithm [14], and the paper by Farrell, Green and Aziz [1] is a great reminder of the clinical utility of DBS in treatment of chronic pain. We applaud the authors for their deep dedication to the spirit of innovation and their timely critical appraisal of cerebral neurostimulation based on unique clinical experience.
